# Eating Patterns in Children with Autism Spectrum Disorder

**DOI:** 10.3390/healthcare10101829

**Published:** 2022-09-21

**Authors:** Danay Ahumada, Barbara Guzmán, Soledad Rebolledo, Karol Opazo, Luis Marileo, Solange Parra-Soto, Sharon Viscardi

**Affiliations:** 1 Carrera de Nutrición y Dietética, Departamento de Procesos Diagnóstico y Evaluación, Facultad de Ciencias de la Salud, Campus San Francisco, Universidad Católica de Temuco, Temuco 4813302, Chile; 2 Programa de Magister en Epidemiología Clínica, Facultad de Medicina, Universidad de la Frontera, Temuco 4813115, Chile; 3 Programa de Doctorado en Ciencias Agropecuarias, Facultad de Recursos Naturales, Universidad Católica de Temuco, Temuco 4813302, Chile; 4 Biotechnology of Functional Foods Laboratory, Camino Sanquilco, Padre Las Casas 4850827, Chile; 5 School of Health & Wellbeing, University of Glasgow, Glasgow G12 8RZ, UK; 6 School of Cardiovascular & Metabolic Health, University of Glasgow, Glasgow G12 8TA, UK; 7 Departamento de Nutrición y Salud Publica, Universidad del Bio-Bio, Chillan 3780000, Chile; 8 Laboratorio de Investigación en Salud de Precisión, Departamento de Procesos Diagnóstico y Evaluación, Facultad de Ciencias de la Salud, Campus San Francisco, Universidad Católica de Temuco, Temuco 4813302, Chile; 9 Núcleo de Investigación en Producción Alimentaria, Universidad Católica de Temuco, Temuco 4813302, Chile

**Keywords:** autism spectrum disorder, eating patterns, food selectivity

## Abstract

The purpose of this research was to analyze the eating patterns of preschool- and school-aged children with ASD, as provided by their families, in the La Araucanía Region of Chile. It involved a cross-sectional study with 72 families with children diagnosed with ASD aged between 2 and 12 years old. Food selectivity, appetite, body mass index (BMI) and frequency of food consumption were studied. The research determined that 97.67% present food selectivity, corresponding to alterations in the frequency of consumption of specific food groups. Moreover, 93.06%, 90.28%, 80.56% and 62.50% of children in the study do not meet the daily recommendations for fruit, fish, water and vegetable consumption, respectively. Therefore, it is important for these findings to be considered when designing and carrying out educational interventions regarding food in families with children with ASD for greater assertiveness and effectiveness in improving health.

## 1. Introduction

Autism spectrum disorder (ASD) is a developmental neurobiological disorder. ASD is a multifactorial condition caused by genetic and environmental factors that generate changes on both sensory and social levels [1,2]. These symptoms arise during the first three years after birth and continue throughout the life cycle. According to the international classification of mental disorders, the fundamental characteristics of ASD include an abnormal or deficient development of social interactions and communication, and the maintenance of a very restricted repertoire of activities and interests, as described in the Diagnostic and Statistical Manual of mental disorders DSM-5, 2013 [3].

The MINSAL Clinical Practice Guide in Chile refers to the detection and timely diagnosis of autism spectrum disorders [4], explaining that there is no centralized registry of how many people have been diagnosed with ASD, nor is there systematic evidence of its prevalence on a national level. Therefore, incidence in Chile is based on global estimations provided by various entities that have historically recorded the prevalence of ASD in children. According to the World Health Organization, 1 out of every 160 children in the world has ASD, and according to the Report of the Centers for Disease Control and Prevention, 1 out of every 59 eight-year-old children in the United States has ASD. On a nationwide level, the Chilean Ministry of Education reports that 1% of the total enrollment registered in educational establishments has identified special needs (by type of disability) corresponding to ASD.

ASD is a disorder that is associated with sensory processing difficulties, which include a heightened or dampened sensitivity to sensory stimuli in the environment [5]. This means that a large number of children with this disorder present some type of problem associated with eating and diet, which is technically known as food selectivity.

Between 46% and 89% of children with ASD are estimated to have dietary problems, ranging from unusual eating patterns (defined as the set of foods or products that an individual or family consumes on a regular basis), rituals and food selectivity. Such characteristics frequently lead to a demand for certain foods, which generates great concern due to its negative impact when trying to achieve an adequate balance of nutrients in family meals [6]. In general, many children with ASD demonstrate atypical eating behaviors during childhood and maintain more limited diets than children without ASD [7]. Food selectivity is thus often reported as a common cause of problems and conflict during mealtimes [8]. This selectivity has been associated with a preference for energy-dense foods, notably sweetened beverages, and a reduced consumption of foods rich in nutrients such as lean protein, fruit, vegetables and high-fiber foods [1].

Food selectivity associated with ASD increases the challenges faced by caregivers of children with this disorder. When trying to offer a healthy diet, they are faced with sensory barriers that, due to lack of knowledge, training or explanatory guides, often trigger malnutrition in their children. Additionally, the decrease in or interruption of meals can lead to an increase in parental stress and a deterioration of the child’s health, causing families to adapt routines at mealtimes [9]. In many cases, caregivers are unable to meet the nutritional recommendations set forth by the Dietary Guide through Adolescence issued by the Chilean Ministry of Health. As a result, training instances for caregivers can often prove to be very useful in cases of moderate food selectivity [10].

To date, however, there are no studies that determine the type of diet that is most common in children with ASD. Therefore, the main aim of this study is to analyze the eating patterns of preschool- and school-aged ASD children in Chile, based on what is provided by their families, with a focus on the La Araucanía region.

## 2. Materials and Methods

### 2.1. Identification of Eating Patterns of Children with ASD, as Provided by Their Families

This study was cross-sectional, composed of a sample of preschool- and school-aged children who live in the region of La Araucanía and who have been diagnosed with ASD, based on the Diagnostic and Statistical Manual of mental disorders DSM-5 by an expert health professional [3]. The sample size was expressed in a non-probabilistic sample for convenience, since there are no records of the ASD population in the country. The parents and/or caregivers of the children in the study agreed to provide data related to the diet maintained by the children and the criteria used to establish this [11]. The families that participated in the study were recruited through different means [12]: therapeutic centers for children diagnosed with ASD, educational establishments, social networks and support groups for parents of ASD children. All were contacted by email, although the most successful means of recruitment was through social media. The following criteria were required for inclusion in the study: first, children aged between 2 and 12 years old diagnosed with ASD must not have another mental and/or neurological disorder such as schizophrenia or refractory epilepsy; second, ASD children must be fed orally, and not through enteral or parenteral methods; and third, ASD children must not be suffering from digestive diseases such as inflammatory bowel disease, chronic diarrhea or infection with *Helicobacter pylori.*

### 2.2. Instrument Used for Evaluating Eating Patterns

This research considered the Institutional Ethics Committee (no. 25/20), and appropriate consent or assent procedures were followed. After participants accepted and signed the study’s informed consent, the instrument itself consisted of a questionnaire of 64 questions divided into 5 dimensions, with discrete nominal, ordinal and quantitative–qualitative variables. It was adapted from published studies, which were chosen based on the following criteria: (1) original publications of observational studies (cohorts, case–control and cross-sectional studies) published between 2010 and 2020; (2) publications written primarily in English; (3) research carried out in a population of children diagnosed with ASD; (4) studies comparing children diagnosed with ASD versus children with TD (typical development). The questionnaire used in our study was adapted from three published surveys: (1) Nutrition Interventions for Children with Special Health Care Needs11; (2) Parents’ motives for food choices and their associations with children’s food preferences: Public Health Nutrition [13]; and (3) Dietary diversity and food intake of urban preschool children in North-Western Sri Lanka: Maternal and child nutrition [11]. The researchers used these studies to gather questions, translate them into Spanish and adapt them to the Chilean population. Prior to the dissemination of the instrument, it was evaluated and validated by the Institutional Ethics Committee of Research. The questions from each survey were selected to characterize the population of children being studied and to identify the eating and/or digestive problems that they present, as well as their behavior when eating. Another item included was the influence on the mood and/or behavior in the child when choosing a snack. Finally, the study looked at the frequency with which the child consumes different food groups.

### 2.3. Study of the Reasons behind Parents’ Motivation to Offer a Certain Eating Pattern

This study is the result of the answers provided in the online questionnaire. To understand the reasons that drive parents to provide or offer certain food to their children, the questionnaire addressed a number of factors. These included eating and/or digestive problems that influence food choices, whether these are food allergies, family decisions or digestive disorders such as vomiting, diarrhea, constipation, among others. Additionally, the study included questions regarding the behavior adopted by the child at mealtimes, the utensils used, the time it takes to eat and the difficulties encountered with food due to its organoleptic characteristics (taste, color, smell and/or texture), given the sensory sensitivity and high food selectivity in ASD children.

To understand the decisions that parents and/or caregivers make when giving a particular food to the child, the questionnaire asked about the main reasons considered when choosing a particular snack, along with the effects of the snack on the child’s mood. The last section of the questionnaire was the food frequency section, where respondents described the daily or weekly frequency with which the child consumes different food groups. This item was expressed in options, where the respondent chose only one from a set of alternatives.

### 2.4. Consumption Frequencies Provided by Parents in Comparison to the Dietary Guide through Adolescence

The study interpreted the data obtained on the frequencies of food consumption on a qualitative level, in terms of the consumption trend indicated by parents based on what they gave their children diagnosed with ASD, and this was compared with recommendations for children in the Dietary Guide through Adolescence issued by the Chilean Ministry of Health [4]. SPSS was used to visualize Spearman’s correlation analysis and categorical principal component analysis (CATPCA).

## 3. Results

The study consisted of the participation of 74 parents and/or caregivers; 72 children were included who met the inclusion criteria mentioned above and 2 respondents were excluded as they did not meet the criteria.

Table 1 presents the characteristics of the population under study disaggregated by gender. The age of the children with ASD in the survey ranged from 2 to 12 years old, and most (43.06%) of these were between 5 and 7 years old. The majority (80.56%) of children with ASD in the study were male, and 19.44% were female.

In terms of geographical location of the population studied, 75% lived in the municipality of Temuco, 15.38% lived in the Padre las Casas municipality and 9.2% lived in the Vilcún municipality, all in the La Araucanía Region in southern Chile. Of those surveyed, 23.61% belong to or identify with the Mapuche ethnic group.

Of the caregivers of children with ASD in the study, 95% are their mothers. The results also indicate that, for all caregivers, 55.56% state that they need help to carry out daily activities and 33.3% state that they require significant help. As a consequence, 33% of the children are seen by three professionals, and 23.61% are seen by two professionals for therapeutic assistance. In terms of the nutritional status of the children, as determined by Body Mass Index (BMI), 63.1% have overnutrition due to excess food intake, 30.9% are in the normal range and 5.88% are undernourished.

### 3.1. Eating Patterns Provided by Families to Children with ASD

The results of the consumption frequency of the different food groups highlighted several key findings. In terms of dairy, 36.11% of the children consume dairy products one to two times a day, whereas 30.56% consume these three times a day. For cereal, 79.17% consume one or two daily portions of cereal; however, 16.67% do not consume these at all. Regarding bread, 63.89% state that their children consume one to two daily servings, and 23.61% consume three to four servings. Questionnaire responses indicate that 55.17% of the children consume one to two servings of vegetables daily; however, 36.11% do not eat this food group at all. Finally, 43.06% of the children consume one daily serving of fruit, and 22.22% consume more than three servings.

When taking a look at the weekly consumption of the food groups, 48.61% of ASD children consume one serving a week of red meat; however, 25.00% do not consume it on a weekly basis. A total of 56.94% consume white meat at least once a week; however, 22.22% never consume white meat. A total of 44.44% of the children do not consume fish regularly, and 40.28% consume fish occasionally (once a week). The situation is similar for eggs: 38.89% of those surveyed reported that the children do not consume eggs, due to preference, whereas 29.17% consume eggs one to two times a week. The study found that the consumption of ultra-processed foods one to two times weekly, such as processed meats and fast food, is 54.17% and 44.44%, respectively. The consumption of sweets or sweet snacks is also prevalent, with 43.06% of children consuming these one to two times a week and 30.56% consuming these more than three times a week.

Regarding the frequency of daily consumption of liquids, 33.33% drink one or two glasses of natural water. For soft drinks, 26.39% drink between one and two glasses a day, 13.89% drink between three and five glasses and 51.39% declare that they do not consume these on a daily basis.

### 3.2. Reasons That Motivate the Eating Patterns Provided by Parents

Of the participants in this study, 50% of ASD children have an increased appetite, reflected in the fact that in consuming their main meals, 47% take between 5 and 15 min (considered fast), whereas 19.44% take longer (Table 2). The most recurrent receptacles used when drinking water, milk and/or milk formula are cups, glasses and especially bottles (used in 37.50% of cases). However, utensils are less frequently used when consuming solid foods. In fact, 62.50% of children prefer eating with their fingers. Consequently, 91.67% of the ASD children surveyed present food selectivity, which is reflected mainly by the organoleptic characteristics of the food, such as its color, taste, smell and texture. Specifically, the most frequently preferred category for each of these characteristics is white (color, 13.89%) and soft or crunchy foods (soft texture: 25%, crunchy texture: 19.44%), whereas for odor, 66.67% indicate no preference for a particular aroma. In ASD, not only is food selectivity evident, but also concomitant disorders may arise, such as pica, which is characterized by repeatedly consuming elements that are not recognized as food. Although 83.33% reported not having this disorder, 4.17% had xylophagia (consumption of paper and wood products) and 4.17% had foliophagy (consumption of leaves).

### 3.3. Comparison of Consumption Frequencies Provided by Parents with the Dietary Guide through Adolescence

A comparison of the frequency of food consumption with the recommendations of the Dietary Guide through Adolescence [4] revealed that 52.78% of children diagnosed with ASD meet the daily dairy recommendation (three servings/day), 90.28% do not meet the fish consumption recommendation (two servings/week), 72.22% comply with meat consumption (three servings/week) and 59.72% comply with weekly egg consumption (two servings/week). Regarding vegetables and fruit, 93.06% and 62.50%, respectively, do not comply with the Dietary Guide through Adolescence. Likewise, 79.17% comply with the daily consumption recommendations for cereal, potatoes and fresh legumes (two servings/day), and 63.89% meet bread intake guidelines. However, 80.56% do not meet the recommendation to consume six to eight glasses of water a day.

**Figure 1:**
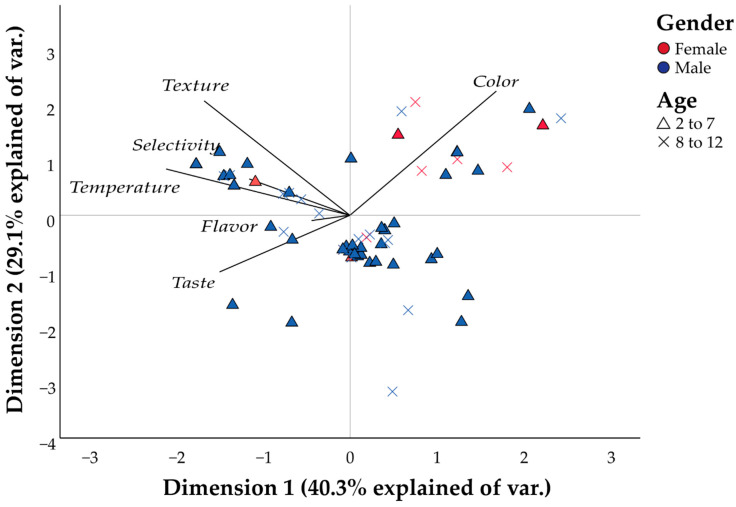
Categorical principal component analysis (CATPCA) based on the preference of children on sensitive attributes of their food (selectivity, color, flavor, taste, texture, temperature).

The study performed a CATPCA to visualize the associations of children’s food preferences with sensory attributes. Figure 1 responds to 40.3% of the variations in dimension 1 and 29.1% of the variations in dimension 2. Color is a sensitive attribute of preference among girls, grouping girls between 5 and 12 years in quadrant I associated with this attribute. Another group of girls evaluated are grouped in quadrant IV. The similarities found among the girls present in quadrant IV, which corresponds mainly to girls aged between 2 and 4 years, are not answered by the attributes evaluated. The groups of children aged between 5 and 7 years, as well as children aged between 11 and 12 years, are associated with quadrant II. Quadrant II is defined by children with similar preferences associated with texture, temperature and some type of food selectivity. Children aged between 2 and 4 years old evaluated in this study present dispersion in preferences in terms of sensitivity, and a large number of children evaluated are grouped in quadrant IV. As with the girls, this trend with quadrant IV indicates that the similarities that children may have in terms of sensitive attributes are not represented by the attributes evaluated.

**Figure 2:**
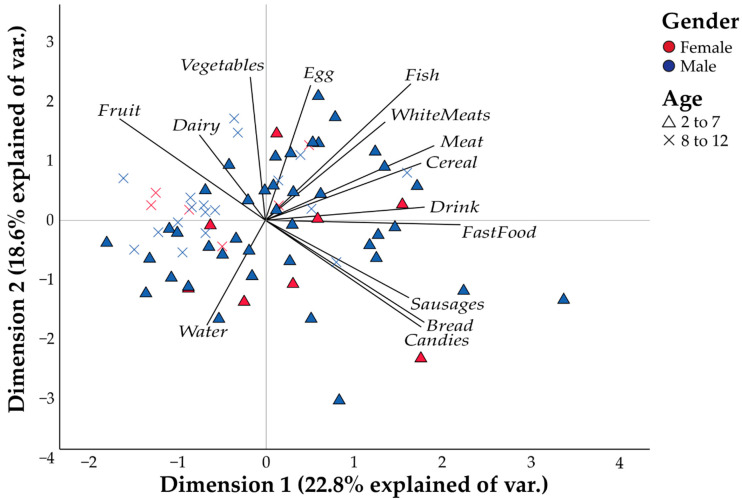
Categorical principal component analysis (CATPCA) based on the preference of children for the food groups evaluated.

Figure 2 reflects the preferences in terms of the food groups evaluated. In this case, there is a general consensus in terms of preferences. Girls and boys aged between 2 and 12 years evaluated in this study are mainly grouped into quadrants II and III, and these quadrants are associated with vegetables, fruit, dairy (quadrant II) and water (quadrant III).

Spearman’s correlation analysis indicates that gender is a significant factor in the preferences for sensitive attributes (Table 3). Odor and selectivity present a significant correlation in terms of the gender of the children evaluated. In terms of age, food preferences present a direct significance with dairy and inverse significance with bread and sausages. Given the nature of the data analyzed, we can infer that as age increases in the children evaluated, the preference for dairy consumption also increases. Likewise, at a younger age, the children evaluated tend to not like dairy or see it as an unfamiliar option.

## 4. Discussion

Autism spectrum disorder is a condition that is accompanied by high food selectivity, and parents of children with ASD have reported that texture, appearance, brand, packaging, temperature, food presentation, color, taste and smell are all characteristics that influence their children’s food choices [14]. Consequently, this study identifies reasons and difficulties related to the eating patterns of ASD children that cause parents to provide certain food groups more frequently [15]. In our study, we analyzed a group of ASD children ranging in age from 2 to 12 years, who reside in the La Araucanía Region of southern Chile. Of the children surveyed, 23.61% belong to or identify with the Mapuche ethnic group. Currently, the population in Chile that is considered to belong to, or feels identified with, an indigenous or native people amounts to 2,185,729 people in the 2017 Census, of whom the Mapuche ethnic group is the most widespread (97.8%).

The study identified food selectivity in 91.67% of the children with ASD, mainly associated with preferences in the organoleptic characteristics of the food. For example, white was the preferred choice (13.89%) in the color category. This demonstrates that the restricted set of interests in children with ASD extends to eating patterns. This can manifest itself in many ways, including, but not limited to, only eating foods of a certain color [16]. Regarding textures, 25% prefer a soft texture on the palate, followed by 23.61% who reported having no particular preference. In terms of smell, 66.67% indicate not having a preference for a specific aroma. However, there are studies that demonstrate that people with ASD report that they prefer familiar food (food neophobia), and do not like food with particular textures and strong flavors [17]. In terms of the childrens’ appetite, 50% have an increased appetite. However, between both variables of food selectivity and appetite there were no significant differences (*p* = 0.693).

Categorical principal component analysis (CATPCA) based on the preference of children on sensitive attributes of their food (Figure 1) shows a 69.4% variance. There is an association between a preference for the color of food among the girls evaluated in the study, where half of the girls were positioned in quadrant I, associated mainly with the color attribute; however, there is no significant correlation between the color and the age or gender of the children evaluated. Children are mainly grouped in quadrant IV, which is not associated with the attributes evaluated. The age of the children presents a significant correlation with the selectivity (Rho = −0.223) and flavor (Rho = −0.273) attributes.

CATPCA based on the preference of children for food groups (Figure 2) shows a 41.4% variance. There is dispersion of the population evaluated in terms of gender, but age is an important variable in terms of the preference for food groups. Age can be a relevant factor in preference for food groups. Age is significantly correlated with dairy (Rho = 0.439), cereal (Rho = −0.225), bread (Rho = −0.360), fast food (Rho = −0.301) and processed meats (Rho = −0.321). The only food group preference associated with gender was fish (Rho = 0.252).

In relation to the gender of the population studied, males predominated (80.56%) over females (19.44%). Boys with ASD have a slightly stronger association between repetitive behaviors, food selection and diagnosed sleep problems than girls [18]. As a result of the nutritional status of ASD children, 63.1% present overnutrition, 30.9% are in the normal range and 5.88% are undernourished. While the association between gender, BMI and appetite is statistically significant with *p* = 0.004 for males, this relationship is not statistically significant for females (*p* = 0.130).

In terms of the predominance of overnutrition in the group surveyed (63.1%), according to several studies [10,15,18,19,20], ASD children are five times more likely to have eating problems than their peers who display typical development. The main difficulty is food selectivity, composed of eating patterns with a preference for processed foods, sandwiches and starches, and a dislike of fruit and vegetables [10]. Such evidence correlates well with the results of our study, which found that 54.17% and 44.44% consume ultra-processed foods, such as processed meats and fast food, respectively, one to two times a week. The study also reveals a substantial consumption of sweets or sweet snacks; 43.06% consume such energy-rich and nutrient-deficient foods one to two times a week, and 30.56% do so at least three times a week. In terms of fruit, 43.06% consume one daily portion, and 22.22% ingest at least three portions. In comparison with the Dietary Guide through Adolescence [4], 62.50% of children with ASD do not comply with the daily recommendation of fruit consumption. Food disturbance and sensory sensitivity are general features of children with neurodevelopmental disorders, including the aversion of children with ASD to fruit [19]. Therefore, given the higher rates of rejection of the consumption of fruit and vegetables, there is a need to adapt the food to the sensory experiences that are to the child’s liking. For example, by physically transforming food into a crunchy texture, it is more acceptable to a greater number of children with ASD [21].

Overall, the higher frequency of gastrointestinal symptoms, food selectivity and difficulties when eating are a common problem in preschoolers and schoolchildren with ASD [19]. Therefore, as food selectivity commonly develops in children with ASD, this can lead to vitamin, mineral and fatty acid deficiencies, such as insufficient intake of vitamin D and calcium. As a result, the majority present a nutritional diagnosis outside the normal range [22].

## 5. Conclusions

The results obtained by the study confirm previous findings by various authors, which show that feeding children with autism spectrum disorder (ASD) involves difficulties in families when choosing what foods to provide. Not only is food selectivity a concern (as determined by preferences for certain organoleptic characteristics), but also behavioral problems are frequently reported when eating, due to the time spent eating and/or the utensils used. Regarding the children’s appetites, 50% have an increased appetite, although there was no correlation between food selectivity and appetite (*p* = 0.693). Consequently, families choose to provide a diet focused on the tastes and/or preferences of the child, meaning that in most cases, children with ASD do not meet the recommendations of the Dietary Guide through Adolescence. For this reason, we recommend ongoing research and evaluation into the behavior and eating patterns of children with ASD, to pinpoint the main areas to consider when nutritionally addressing these children and their families, and using this to guide nutritional and other professionals in encouraging caregivers to make improvements in diet starting in early childhood.

## Figures and Tables

**Table 1:** Characterization of the population studied according to the gender of the child. The descriptive analysis of the participants includes age, Body Mass Index (BMI) and the degree of help required.

Age	2 to 7				8 to 12					
Gender	Female		Male		Female		Male		Total	
	*n*	(%)	*n*	(%)	*n*	(%)	*n*	(%)	*n*	(%)
**BMI**										
*Undernourished*	1	(1.4)	1	(1.4)		−		−	2	(2.8)
*Risk of undernourishment*		−		−		−	2	(2.8)	2	(2.8)
*Normal*	3	(4.2)	12	(16.7)		−	6	(8.3)	21	(29.2)
*Overweight*	3	(4.2)	6	(8.3)		−	3	(4.2)	12	(16.7)
*Obese*	2	(2.8)	11	(15.3)	3	(4.2)	5	(6.9)	21	(29.2)
*Severely Obese*		−	6	(8.3)	1	(1.4)	3	(4.2)	10	(13.9)
*Not refer*									4	(5.6)
**Help level**										
*Needs help*	5	(6.9)	21	(29.2)	1	(1.4)	13	(18.1)	40	(55.6)
*Needs significant help*	5	(6.9)	12	(16.7)	1	(1.4)	6	(8.3)	24	(33.3)
*Needs very significant help*		−	5	(6.9)	2	(2.8)	1	(1.4)	8	(11.1)

**Table 2:** Reasons that motivate parents to choose an eating pattern.

Age	2 to 7				8 to 12					
Gender	Female		Male		Female		Male		Total	
	*n*	(%)	*n*	(%)	*n*	(%)	*n*	(%)	*n*	(%)
**Child’s appetite**										
*Very little appetite*	1	(1.4)	2	(2.8)		−	1	(1.4)	4	(5.6)
*Little appetite*		−	8	(11.1)		−		−	8	(11.1)
*Normal appetite*	4	(5.6)	9	(12.5)		−	11	(15.3)	24	(33.3)
*Large appetite*	5	(6.9)	19	(26.4)	4	(5.6)	8	(11.1)	36	(50.0)
**Time it takes to eat**										
*5−15 minutes*	5	(6.9)	17	(23.6)	2	(2.8)	10	(13.9)	34	(47.2)
*15−20 minutes*	2	(2.8)	8	(11.1)	2	(2.8)	5	(6.9)	17	(23.6)
*20−30 minutes*		−	5	(6.9)		−	2	(2.8)	7	(9.7)
*More than 30 minutes*	3	(4.2)	8	(11.1)		−	3	(4.2)	14	(19.4)
**Eating disorder**										
*Geophagy (soil)*		−	1	(1.4)		−		−	1	(1.4)
*Xylophagy (wood)*	2	(2.8)	1	(1.4)		−		−	3	(4.2)
*Onychophagia (nails)*		−	1	(1.4)		−		−	1	(1.4)
*Foliophagia (paper)*	1	(1.4)	1	(1.4)	1	(1.4)		−	3	(4.2)
*Yes, but does not specify*		−	3	(4.2)		−	1	(1.4)	4	(5.6)
*Does not consume items other than food*	7	(9.7)	31	(43.1)	3	(4.2)	19	(26.4)	60	(83.3)

**Table 3:** Association between population segmentation (gender and age) and assessed food preferences through Spearman’s correlation. * Indicates that the correlation is significant at the 0.05 level (bilateral).

	Age	Gender
**Sensitive attribute**		
*Selectivity*	−0.213	**−0.233** *
*Color*	−0.032	−0.032
*Flavor*	0.063	**−0.273** *
*Taste*	−0.110	0.073
*Texture*	−0.150	0.005
*Temperature*	−0.135	−0.015
**Food group**		
*Dairy*	**0.430** *	0.007
*Cereal*	−0.208	0.168
*Bread*	**−0.289** *	−0.055
*Vegetables*	0.071	−0.048
*Fruit*	0.137	−0.116
*Meat*	−0.072	0.029
*White meats*	0.083	0.192
*Fish*	−0.144	**0.252** *
*Egg*	−0.074	−0.012
*Fast food*	−0.183	−0.092
*Sausages*	**−0.247** *	0.081
*Candies*	−0.003	−0.148
*Water*	−0.027	0.142
*Drink*	−0.157	0.105

## Data Availability

Not applicable.
